# Valuable prognostic indicators for severe burn sepsis with inhalation lesion: age, platelet count, and procalcitonin

**DOI:** 10.1186/s41038-018-0132-1

**Published:** 2018-10-29

**Authors:** Yichao Xu, Xinyuan Jin, Xiaonan Shao, Feng Zheng, Hong Zhou

**Affiliations:** 1 Department of Burns, Fujian Medical University Union Hospital, Fuzhou, 350000 China; 2 Department of Burn Plastic Surgery, The Affiliated Suzhou Hospital of Nanjing Medical University, Suzhou, 215008 China; 3 Department of Nuclear Medicine, The Third Affiliated Hospital of Soochow University, Changzhou, 213003 China; 4 Department of Critical Care Medicine, The Third Affiliated Hospital of Soochow University, Changzhou, 213003 China; 5 Department of Plastic Surgery and Burns, The Third Affiliated Hospital of Soochow University, Changzhou, 213003 China

**Keywords:** Burns, Sepsis, Age, Platelet count, Procalcitonin

## Abstract

**Background:**

Severe burn sepsis can lead to high mortality. We explored the valuable prognostic indicators for severe burn sepsis with inhalation lesion.

**Methods:**

Thirty-eight severe burn patients with sepsis who were admitted to the Third Affiliated Hospital of Soochow University from August 2014 to December 2017 were retrospectively analyzed. Among them, 22 patients were assigned to the death group and 16 patients to the survival group. Their general information, vital signs, and blood index including serum procalcitonin (PCT) and C-reactive protein (CRP) levels at admission, diagnosis of sepsis, and 1-week post-diagnosis of sepsis were compared.

**Results:**

Patients in the death group were older and had lower platelet count (PLT) at diagnosis of sepsis and 1-week post-diagnosis as well as higher PCT level at 1-week post-diagnosis than patients in the survival group (all *p* < 0.05). According to receiver operating characteristic (ROC) curves, the above four indicators could be used to predict the prognosis, and the area under the curve (AUC) of PLT at diagnosis and 1-week post-diagnosis was larger (0.888 and 0.911), and PLT at diagnosis had the highest sensitivity and specificity (0.842 and 0.937). In addition, these patients were divided into two groups by the optimal cutoff age of 38 years. According to multivariate logistic regression analysis and COX regression analysis, only age group and PLT at diagnosis were statistically significant (all *p* < 0.05). The risk of death in the older group was 28 times higher than that in the younger group, and the risk of death increased by 3% for each unit reduction in PLT at diagnosis. Moreover, age group was an independent factor affecting the patients’ survival (*β* = − 1.370, *p* = 0.026). Considering the survival time after sepsis, the mortality risk was lower for patients in the older group than for patients in the younger group (hazard ratio (HR) = 0.254, 95% confidence interval (CI) 0.076–0.851).

**Conclusion:**

Age, PLT at diagnosis of sepsis, and 1-week post-diagnosis as well as PCT level at 1-week post-diagnosis are indicators for the evaluation of prognosis of severe burn sepsis with inhalation lesion. Among them, PLT at diagnosis has the greatest prognostic value. In addition, age can predict the patients’ mortality and survival time after sepsis.

## Background

Unlike other wounds, severe burns can lead to more intense and lasting systemic inflammatory response syndrome (SIRS) and a higher incidence of whole body infection and sepsis due to skin barrier damage and organ dysfunction [1]. Its further development can lead to septic shock and multiple organ dysfunction syndrome (MODS) [2] among others. A recent study demonstrated that mortality in patients with sepsis after burns was 34.4% [3]. Currently, burn sepsis has become the main obstacle for the treatment of severe burns [4].

Blood cultures are still the “golden standards” for definitive sepsis identification, but they require 48–72 h and are unable to rapidly diagnose sepsis in burn patients. In addition, because of the usage of high-dose antibiotics at an early stage in the clinic, the positive detection rate of blood culture is very low, which would delay the diagnosis. Moreover, blood culture is vulnerable to external bacterial contamination, leading to misdiagnosis [5]. At present, the indexes for clinical diagnosis of sepsis of burn patients, such as fever, white blood cells (WBC), increased neutrophil percentage (*N*%), thrombocytopenia, tachycardia, and tachypnea, all have a role in the determination of patients’ condition [6]. Although these indexes are easy to monitor, they are not sensitive enough to infection. C-reactive protein (CRP) is an acute phase protein and considered as an indicator of early diagnosis of inflammation [7]. But besides the infection, CRP is also affected greatly by many other conditions such as trauma, surgery, burns, tissue necrosis, immune-mediated inflammatory disease, and advanced cancers. Patients with severe burns do have a systematic inflammatory response (SIR). Therefore, it is very important to develop new methods for differential diagnosis between a pure inflammatory reaction and a true sepsis, due to microbiological invasion of the bloodstream [8].

Previous reviews have shown that procalcitonin (PCT) may be used as an auxiliary index in clinical diagnosis of sepsis and a modality to reduce exposure of antibiotics to critically ill patients [9] and may be the most promising biomarker of burn patients with sepsis [10, 11]. Reports on the evaluation of prognostic indexes of severe burn sepsis with inhalation lesion are rare. Thus, the present study was designed to compare the changes in indexes and analyze their values in the prognosis of severe burn patients with sepsis.

## Methods

### Inclusion criteria and exclusion criteria

Patients were enrolled in the study if they met the following criteria: (1) had burn area > 30% total body surface area (TBSA) [12] and burn grade of II–III, (2) ≥ 18 years old, (3) moderate to severe inhalation injury was diagnosed by clinical exam and bronchoscopy at admission [13], and (4) sepsis was diagnosed based on the American Burn Association criteria [6]. Patients were excluded if they met the following exclusion criteria: (1) had negative blood culture during the length of stay and (2) died of acute pulmonary edema or non-septic shock.

### Clinical information

The study was approved by the Ethics Committee of the Third Affiliated Hospital of Soochow University. All patients and their family members signed the informed consent form. Patients’ medical records were anonymous. All patients including 30 males and 8 females were admitted to the Third Affiliated Hospital of Soochow University within 12 h of accident from August 2014 to December 2017. They were at the age of 20–65 years old and given comprehensive treatments including anti-shock, anti-infection, protection of internal organ function, debridement, and early deep wound surgery as well as tracheotomy for patients with respiratory tract burns. Early application of blood purification was used for clearance of inflammatory mediators and the recovery of the patient’s immune status, especially for those with a greater burn area (≥ 80% TBSA) [14]. Patients with sepsis all had abdominal distension, bowel sounds weakened or disappeared, and the wounds were moist, dull, and with necrotic spot, and so on.

In addition, patients with sepsis were diagnosed at 6–31 days after injury. Among the 38 patients, 22 (57.9%) died at 4–51 days post-diagnosis of sepsis, and 16 survived. For the 16 surviving patients, their conditions were improved after treatment and did not meet the diagnostic criteria for sepsis for three continuous days.

### Observation indexes

Vital signs, blood tests, serum sodium (Na^+^), glucose (Glu), CRP, and serum CO_2_ partial pressure (PaCO_2_) were recorded and measured every day after admission. Serum PCT levels were serially collected immediately after admission and in 3-day periods until discharge or death by automatic fluorescence immunoassay analyzer (Roche cobas8000), and its value of 0–0.5 ng/mL was considered as normal. CRP was measured on BECKMAN COULTER AU5800, and its value in the range of 0–3 mg/L was considered as normal. If multiple blood samples were measured within 1-week post-diagnosis of sepsis, their average was used.

### Statistical analysis

Data were analyzed using SPSS 13.0 software. Measurement data with normal distribution were expressed as mean ± standard deviation (mean ±SD). Measurement data with non-normal distribution were expressed as median (25–75 centiles). Count data were expressed in number or percentage. Normally distributed data between the two different groups were compared using *t* test, and differences in rates were compared using the *χ*^2^ test. Two sets of data with abnormal distribution were compared using the Mann-Whitney *U* rank sum test. Receiver operating characteristic (ROC) curves and area under the curve (AUC) were used to compare the prognosis efficacy of different indexes. The most accurate cutoff value was calculated by the Youden Index. Logistic regression analysis was applied to perform the univariate and multivariate analysis and calculate the regression coefficient and the odds ratio (OR). COX regression analysis was used to compare the influence of different indexes on survival time and calculate the hazard ratio (HR) and 95% confidence interval (CI). A value of *p* < 0.05 was considered statistically significant.

## Results

### General comparison of the patients

There was no significant difference between the two groups in sex ratio, total burn area, area of III degree, and the time of diagnosis of sepsis (all *p* > 0.05). Patients in the death group were significantly older than patients in the survival group (*p* < 0.05), as shown in Table 1.

**Table 1:** General comparison of patients in the death and survival groups

Group	N	Gender		Age (years)	Burn area (%TBSA)		Time of diagnosis of sepsis (days)
		Male	Female		Total area	Area with grade III burn	
Death	22	18	4	43 ± 12	92 ± 7	71 ± 22	12 ± 6
Survival	16	12	4	34 ± 10	89 ± 11	69 ± 20	15 ± 8
*P*		0.698		0.039	0.410	0.770	0.130

Measurement data with normal distribution were expressed as mean ± standard deviation

*TBSA* total body surface area

### Comparison of all indexes at admission, diagnosis of sepsis, and 1-week post-diagnosis

Comparison of all indexes at admission showed that there were no significant differences between patients in the death group and in the survival group (all *p* > 0.05), as shown in Table 2. Comparison of all indexes at diagnosis of sepsis showed that platelet count (PLT) in the death group was significantly lower than that in the survival group (*p* < 0.05), as shown in Table 3. Comparison of serum WBC, *N*%, PLT, PCT, and CRP at 1-week post-diagnosis showed that PLT and PCT were significantly different (all *p* < 0.05) between patients in the death group and in the survival group, as shown in Table 4.

**Table 2:** Comparison of indexes at admission between the death and survival groups

Group	Max. body temp (°C)	Min. body temp (°C)	Heart rate (beats/min)	WBC (× 10^9^/L)	*N*%	PLT (× 10^9^/L)	Na^+^(mmol/L)	Glu (mmol/L)	PCT (ng/mL)	CRP (mg/L)	PaCO_2_(mmHg)
Death	37.1 ± 0.9	36.4 ± 1.1	131 ± 16	31.49 ± 15.25	86.5 ± 6.0	200 ± 122	140.8 ± 6.9	8.99 ± 3.47	10.6 (4.9, 21.4)	23.9 (9.3, 27.7)	39.7 ± 8.0
Survival	37.2 ± 0.9	36.3 ± 0.5	126 ± 24	24.79 ± 13.56	85.6 ± 5.4	172 ± 139	139.0 ± 5.6	8.28 ± 3.85	4.6 (0.5, 9.2)	8.5 (2.2, 25.1)	35.2 ± 5.3
							0.935	0.492	1.470	1.610	1.667
*P*	0.869	0.723	0.490	0.193	0.657	0.545	0.357	0.627	0.156	0.108	0.107

Measurement data with normal distribution were expressed as mean ± standard deviation. Measurement data with non-normal distribution were expressed as median (25–75 centiles)

*WBC* white blood cells, *N%* neutrophil percentage, *PLT* platelet count, *Na*^*+*^ serum sodium, *Glu* glucose, *PCT* procalcitonin, *CRP* C-reactive protein, *PaCO*_*2*_ serum CO_2_ partial pressure

**Table 3:** Comparison of indexes at diagnosis of sepsis between the death and survival groups

Group	Max. body temp (°C)	Min. body temp (°C)	Heart rate (beats/min)	WBC (× 10^9^/L)	*N*%	PLT (× 10^9^/L)	Na^+^(mmol/L)	Glu (mmol/L)	PCT (ng/mL)	CRP (mg/L)	PaCO_2_(mmHg)
Death	38.7 ± 1.6	36.7 ± 1.3	125 ± 23	13.35 ± 6.81	87.9 ± 7.4	94 ± 63	146.0 ± 9.9	10.00 ± 6.64	3.1 (1.2, 8.6)	24.8 (24.3, 26.3)	38.2 ± 8.0
Survival	39.3 ± 0.7	36.8 ± 0.9	137 ± 11	15.34 ± 6.42	87.6 ± 4.3	256 ± 126	143.1 ± 12.2	9.84 ± 6.22	1.3 (0.8, 4.3)	23.4 (17.6, 24.7)	36.4 ± 6.5
*P*	0.236	0.768	0.080	0.382	0.892	0.000	0.427	0.948	0.172	0.079	0.528

Measurement data with normal distribution were expressed as mean ± standard deviation. Measurement data with non-normal distribution were expressed as median (25–75 centiles)

*WBC* white blood cells, *N%* neutrophil percentage, *PLT* platelet count, *Na*^*+*^ serum sodium, *Glu* glucose, *PCT* procalcitonin, *CRP* C-reactive protein, *PaCO*_*2*_ serum CO_2_ partial pressure

**Table 4:** Comparison of indexes at 1-week post-diagnosis of sepsis between the two groups

Group	WBC (× 10^9^/L)	*N*%	PLT (× 10^9^/L)	PCT (ng/mL)	CRP (mg/L)
Death	11.26 ± 4.96	86.5 ± 5.4	97 ± 72	4.3 (2.3, 8.7)	24.2 (23.9, 24.6)
Survival	11.03 ± 2.30	83.0 ± 5.4	268 ± 113	2.2 (1.0, 4.1)	24.4 (24.0, 28.4)
	0.125	1.494	− 4.351	2.500	1.016
*P*	0.902	0.151	0.000	0.011	0.371

Measurement data with normal distribution were expressed as mean ± SD. Measurement data with non-normal distribution were expressed as median (25–75 centiles)

*WBC* white blood cells, *N%* neutrophil percentage, *PLT* platelet count, *PCT* procalcitonin, *CRP* C-reactive protein

### Analyses of indexes using ROC curves

**Fig. 1:**
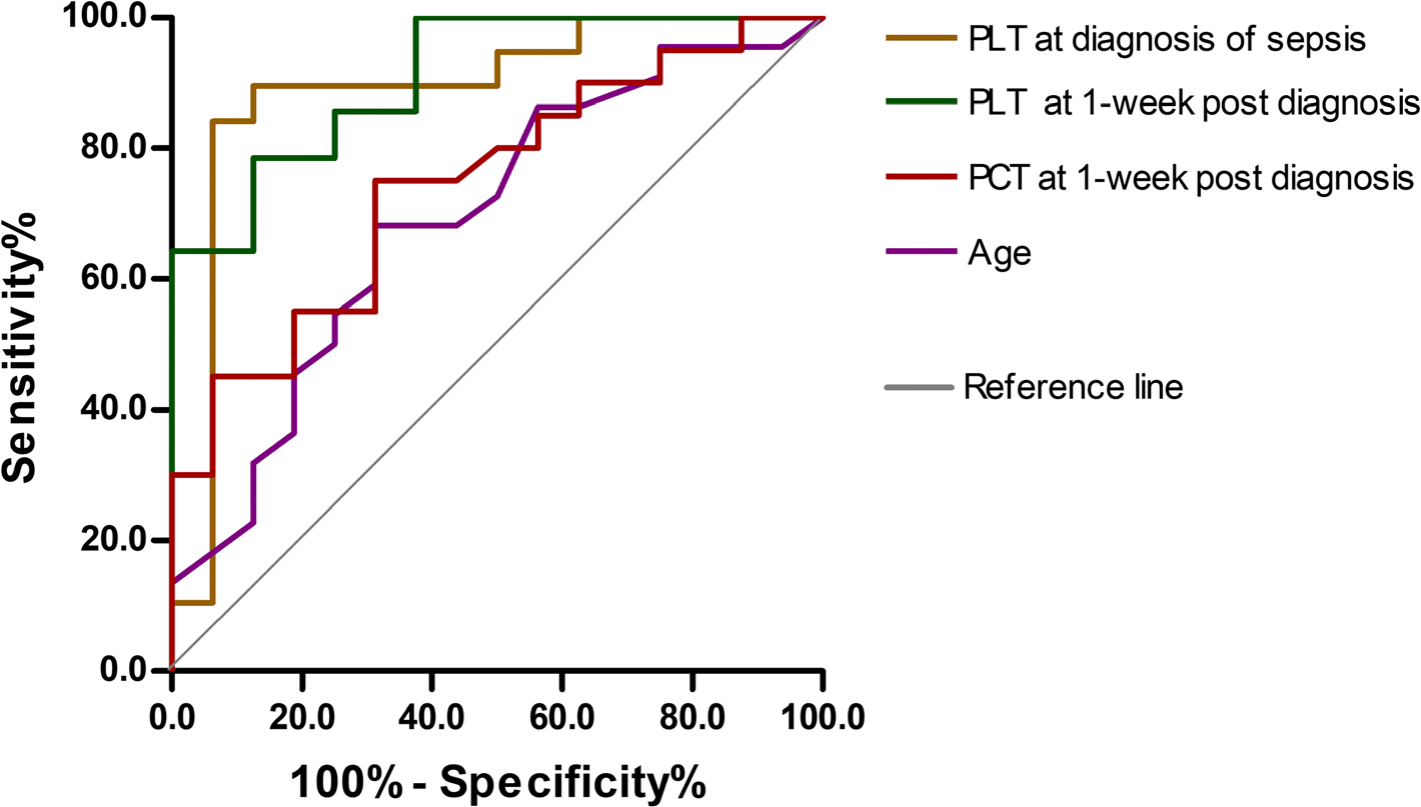
Receiver operating characteristic (ROC) curves of prognosis of severe burn sepsis by age, platelet count (PLT) at diagnosis of sepsis, PLT and procalcitonin (PCT) at 1-week post-diagnosis. The area under the curve (AUC) of PLT at diagnosis and 1-week post-diagnosis was larger (0.888 and 0.911)

ROC curves showed that age, PLT at diagnosis, and 1-week post-diagnosis, and PCT at 1-week post-diagnosis could be used to predict the prognosis of severe burn sepsis (all *p* < 0.05), as shown in Fig. 1 and Table 5. The AUC of PLT at diagnosis and 1-week post-diagnosis were larger (0.888 and 0.911), and PLT at diagnosis had the highest sensitivity and specificity (0.842 and 0.937), followed by PCT at 1-week post-diagnosis and age. The best cutoff value for age was 38 years old; therefore, patients were divided into ≥ 38 years old group (20 cases) and < 38 years old group (18 cases).

**Table 5:** Prognostic value of various indexes in severe burn patients with sepsis by receiver operating characteristic (ROC) curves

Indexes	AUC	*P*	95% CI	Cutoff	Sensitivity	Specificity
Age (years)	0.673	0.044	0.522–0.865	38	0.682	0.687
PLT at diagnosis of sepsis (× 10^9^/L)	0.888	0.000	0.762–1.014	150	0.842	0.937
PLT at 1-week post-diagnosis of sepsis (× 10^9^/L)	0.911	0.002	1.790–1.031	161	0.786	0.875
PCT at admission (ng/mL)	0.700	0.142	0.455–0.945	10.3	0.444	0.900
PCT at diagnosis of sepsis (ng/mL)	0.652	0.104	0.448–0.857	2.0	0.667	0.643
PCT at 1-week post-diagnosis of sepsis (ng/mL)	0.745	0.012	0.586–0.905	2.4	0.750	0.697

*AUC* area under the curve, *CI* confidence interval, *PLT* platelet count, *PCT* procalcitonin

### Logistic regression analysis and COX regression analysis

**Fig. 2:**
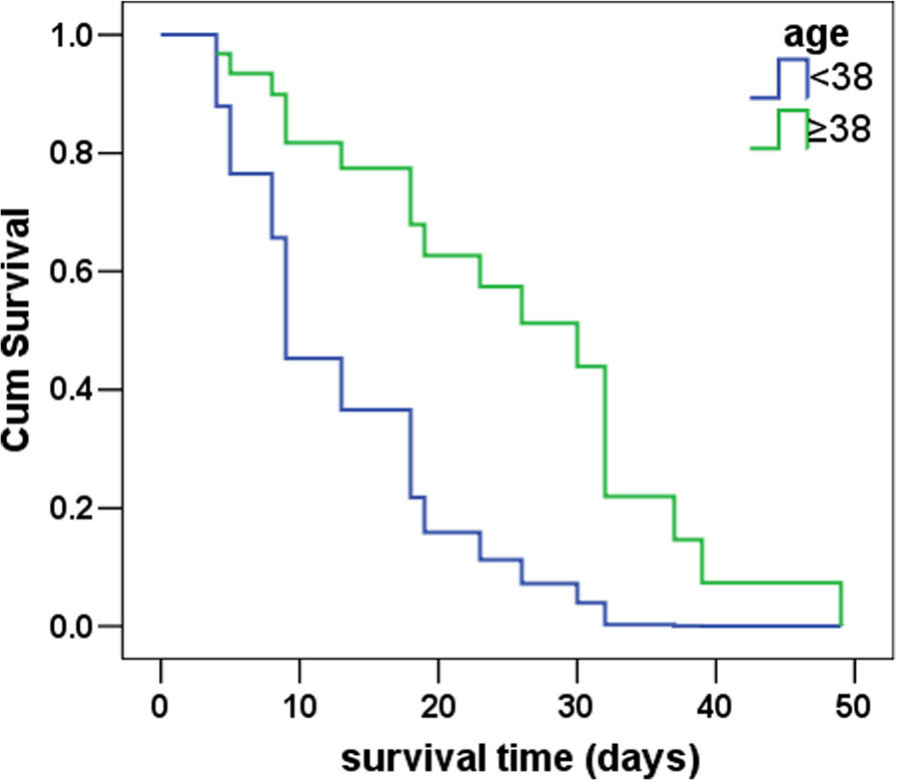
Age-based survival curves with a cutoff of 38 years old. COX regression analysis showed that age group was an independent factor affecting survival (*β* = − 1.370, *p* = 0.026). Considering survival time after sepsis, the mortality risk in the older group was lower than that in the younger group (hazard ratio = 0.254, 95% confidence interval 0.076–0.851)

Univariate logistic analysis showed that the heart rate and CRP at diagnosis were not statistically significant between the two groups (all *p* > 0.05). In addition, considering that PLT at 1-week post-diagnosis had a good correlation with PLT at diagnosis (*r* = 0.872, *p* < 0.05) and PCT at 1-week post-diagnosis (*r* = − 0.563, *p* < 0.05) as well as clinical significance of PLT at diagnosis, age group, PLT at diagnosis, and PCT at 1-week post-diagnosis were included in the multivariate logistic regression analysis and COX regression analysis. Among the three variables included in the logistic model, only the age group and PLT at diagnosis were statistically significant (all *p* < 0.05), as shown in Table 6. The risk of death in the older group was 28 times higher than that in the younger group, and the risk of death increased by 3% for each unit reduction in PLT at diagnosis. COX regression analysis showed that the age group was an independent factor affecting survival (*β* = − 1.370, *p* = 0.026). Considering the survival time after sepsis, the mortality risk in the older group was lower than that in the younger group (HR = 0.254, 95% CI 0.076–0.851), as shown in Fig. 2.

**Table 6:** Logistic regression analysis of the impact of various factors on prognosis

Factors	*β*	*P*	OR	95% CI
Age group (≥ 38 years)	3.347	0.020	28.425	1.688–478.716
PLT at diagnosis of sepsis (× 10^9^/L)	− 0.027	0.007	0.973	0.954–0.992

*OR* odds ratio, *CI* confidence interval, *PLT* platelet count

## Discussion

### Mortality of severe burn patients with sepsis

A previous study has shown that the degree of inhalation injury is related to the burn-related mortality [15]. In addition, regardless of the degree of burns, burn patients with severe inhalation injury are directly diagnosed as severe burns [16]. Several retrospective clinical studies have shown that the mortality of severe burn patients with sepsis can be as high as 58.9% [17]. In our study, the mortality of patients with sepsis was 57.9%, similar to that in the literature. High mortality suggests that early diagnosis and aggressive treatment including early tracheotomy should be adopted to improve the survival of patients.

### Age, sepsis mortality, and survival time

A previous report has shown that age has a positive linear correlation with the mortality of burn patients [18]. Our study also showed that patients in the death group were significantly older. ROC curves indicated that the best cutoff age was 38 years old. Because most of the enrolled patients were young, the optimal cutoff age was low. In addition, age was an independent factor affecting the survival time after sepsis. These results were interesting and indicated that although older patients had higher mortality after severe burn sepsis, they had longer survival time after sepsis than younger patients. Burn patients often have chronic baseline inflammation responses [19] and immune disorders [1]. However, SIR is weaker in older patients than in younger patients [20], so older patients may be more resistant to septic shock or MODS.

### Clinical symptoms and common indexes for prognosis

Although the clinical symptoms and common indexes of burn patients such as body temperature, and heart rate can be easily measured and have some relevance to infection, they cannot be used as sensitive indexes of infection [8]. Increase in WBC or *N*% is one of the diagnostic criteria for burn patients with sepsis [6]. But WBC is affected by many other factors such as levels of catecholamine and glucocorticoid, acute hemorrhage, myocardial infarction, and the like. Thus, WBC is of little significance for diagnosis of infection or sepsis. An increase in WBC in burn patients is not a direct evidence for the diagnosis of infection [13]. Although Na^+^ and Glu have been used for diagnosis of burn patients with sepsis [6], our results indicated that they had limited prognostic values.

It has been reported that CRP level was increased in 12–24 h of pathogenesis, maintained at high level in 20–72 h, and returned to baseline in 3–7 days [21]. In addition, growing evidences have indicated that CRP is not only an inflammatory marker but also directly involved in the inflammatory process itself. Dehne et al. investigated CRP level in 24 cases of severe burn patients and the effects of TBSA on CRP level [12]. They found that CRP level continuously rose in patients with TBSA > 30%. Wu et al. confirmed that CRP levels were not significantly higher among burn patients with septic shock or bloodstream infection [13]. Our results showed that CRP level was not significantly different between the two groups, indicating it had limited prognostic value.

### PLT and prognosis

Thrombocytopenia (< 100 × 10^9^/L) is just one of the sums of clinical criteria used for sepsis diagnosis [6] and has no value when used alone. Thrombocytopenia occurs in 35–59% of patients with sepsis and in 79.6% of patients with positive blood culture. In addition, thrombocytopenia is a dangerous sign of risk of death in patients with sepsis [22]. The recovery of minimal PLT is associated with the reduced mortality [23]. Marck et al. found that PLT reached its minimum 3 days after burn and recovered to its peak 15 days after injury [24]. Patients might have temporary thrombocytosis. During this process, affected by the burn area, age, and sepsis, the area of severe burns, elder age, and low PLT peak count can be used to predict mortality. In this study, the best cutoff PLT at diagnosis of sepsis for mortality prediction of burn patients with sepsis was 150 × 10^9^/L. The reason for such a high cutoff was that sepsis was diagnosed around the peak PLT proposed by Marck et al. [24]. In addition, multivariate analysis suggested that PLT at diagnosis was an independent protective factor affecting prognosis, consistent with the results of ROC.

### Serum PCT level and prognosis

Serum PCT level is closely related to the severity of infection. Higher serum PCT level prompts more severe condition of patients. The continuous increase in serum PCT suggests high mortality of patients [25]. The decrease in post-treatment serum PCT level reflects the effectiveness of anti-infection treatment. Effective treatment and infection control could lead to the rapid restoration of PCT to the normal level [26]. This may be due to that the severity of sepsis depends on SIR caused by a serious bacterial infection. The greater the response, the higher the mortality [27]. Therefore, serum PCT level not only has the effect on the development of burn sepsis but also can predict the prognosis [28], thus is of important guiding significance for implementation of clinical intervention as soon as possible [29].

Kim et al. reported that serum PCT level within the first 48 h after burn, especially within the first 14–24 h after burn, was a useful prognostic indicator for mortality of patients with sepsis [30]. In this study, serum PCT level at 1-week post-sepsis was significantly different between the two groups. Its optimal cutoff value for mortality prediction was 2.4 ng/mL. Wang et al. reported that the cutoff of PCT at diagnosis of sepsis for mortality prediction was 10.9 ng/mL [29]. This discrepancy is possibly due to the following reasons. Firstly, burn patients in different studies may have different clinical manifestations such as burn area, burn type, inhalation injury, and other complications. Secondly, these studies may differ in sample size, which may affect the reliability of the results [10]. Thirdly, PCT measurements from different instruments show variability. Finally, blood samples may be collected at different times. Because after bacterial toxins entering the bloodstream, serum PCT level increases within 3–6 h and reaches its peak within 6–22 h. In addition, PCT has a long half-life (25–30 h); thus, its serum concentration can maintain increasing within 12–48 h [31]. Therefore, PCT level is different in septic shock peak period from that in the recovery period. Therefore, tracking the trend of PCT level after sepsis is of importance to understand the prognosis of patients with severe burn sepsis.

## Conclusions

In conclusion, age, PLT at diagnosis of sepsis, and 1-week post-diagnosis as well as PCT level at 1-week post-diagnosis are indicators for the prognosis of severe burn sepsis with inhalation lesion. Among them, PLT at diagnosis has the greatest prognostic value. In addition, age can predict patients’ mortality and survival time after sepsis. Application of these indexes to timely assess the patients’ prognosis may be of guiding significance for clinical intervention. However, the study was only a single-center retrospective study and had some limitations. Thus, the conclusion of the study needs to be further studied with a larger sample size and at different treatment centers.

## Abbreviations

AUC: Area under the curve
CI: Confidence interval
CRP: C-reactive protein
Glu: Glucose
HR: Hazard ratio
MODS: Multiple organ dysfunction syndrome
Na^+^: Serum sodium
OR: Odds ratio
PaCO_2_: Serum CO_2_ partial pressure
PCT: Procalcitonin
PLT: Platelet count
SIR: Systematic inflammatory response
SIRS: Systemic inflammatory response syndrome
WBC: White blood cells
